# A latent code based multi-variable modulation network for susceptibility mapping

**DOI:** 10.3389/fnins.2023.1308829

**Published:** 2023-12-21

**Authors:** Weibin Zhou, Jiaxiu Xi, Lijun Bao

**Affiliations:** Department of Electronic Science, Xiamen University, Xiamen, China

**Keywords:** deep learning, quantitative susceptibility imaging, modulated convolution, information fusion, cerebral hemorrhage and calcification

## Abstract

Quantitative susceptibility mapping (QSM) is a technique for obtaining quantitative information on tissue susceptibility and has shown promising potential for clinical applications, in which the magnetic susceptibility is calculated by solving an ill-posed inverse problem. Recently, deep learning-based methods are proposed to address this issue, but the diversity of data distribution was not well considered, and thus the model generalization is limited in clinical applications. In this paper, we propose a Latent Code based Multi-Variable modulation network for QSM reconstruction (LCMnet). Particularly, a specific modulation module is exploited to incorporate three variables, i.e., field map, magnitude image, and initial susceptibility. The latent code in the modulated convolution is learned from feature maps of the field data using the encoder-decoder framework. The susceptibility map pre-estimated from simple thresholding is the constant input of the module, thereby enhancing the network stability and accelerating training convergence. As another input, multi-level features generated by a cross-fusion block integrate the information of field and magnitude data effectively. Experimental results on *in vivo* human brain data, challenge data, clinical data and synthetic data demonstrate that the proposed method LCMnet can achieve outstanding performance on accurate susceptibility measurement and also excellent generalization.

## 1 Introduction

Magnetic susceptibility is an intrinsic physical property of a material that reflects its degree of magnetization. For instance, the susceptibility in brain imaging is dominated by iron content, myelin, calcification, hemorrhage etc. (Buch et al., 2015; Champagne et al., 2021). Accurate reconstruction of tissue susceptibility map therefore may provide valuable information for the diagnosis of intracerebral bleeding and calcification, and also for monitoring neuro-degenerative diseases such as Parkinson’s disease, Alzheimer’s disease, multiple sclerosis, Huntington’s disease and even cognitive development in children (Chen et al., 2014; Barbosa et al., 2015; Murakami et al., 2015; Li et al., 2016). Quantitative susceptibility mapping (QSM) is a novel magnetic resonance imaging (MRI) technique that measures the spatial distribution of susceptibility from phase signal of MRI data (Schweser et al., 2013; Deistung et al., 2017). QSM can be applied to *in vivo* tissues, organs and objects (Acosta-Cabronero et al., 2013; Sharma et al., 2015), displaying a broad and promising prospect for scientific researches and clinical applications.

QSM utilizes MRI phase contrast to obtain the local field variation, from which the susceptibility map can be reconstructed by solving the field-to-source inverse problem. Due to singular angles in the dipole kernel, the susceptibility reconstruction is an ill-posed inverse problem (Wang and Liu, 2015). By exploiting the data redundancy, calculation of susceptibility through multiple orientation sampling (COSMOS) can achieve an accurate reconstruction (Liu et al., 2009), and it often acts as the golden standard in result evaluation. However, the multi-orientation scan is time consuming and inconvenient clinically, for it requires the subject to repeatedly change the head orientation. In the single-orientation QSM, thresholded k-space division (TKD) is the simplest and fastest approach, but yields streaking artifacts and misestimating (Wharton et al., 2010). For more accurate measurement, optimization-based methods are proposed with prior regularization constraints, e.g., MEDI (Liu et al., 2012), STAR-QSM (Wei et al., 2015) and SFCR (Bao et al., 2016).

With the great success of deep learning, convolutional neural networks (CNN) have been demonstrated as a powerful tool for medical image processing (Chlemper et al., 2017; Piantadosi et al., 2020; Gravina et al., 2021). At present, CNN is introduced into the susceptibility reconstruction and there are some preliminary attempts on deep learning based QSM (Yoon et al., 2018; Bollmann et al., 2019; Polak et al., 2020; Gao et al., 2021; Si et al., 2023). Based on the U-Net architecture, QSMnet is trained to learn an end-to-end mapping from the field data to the susceptibility map (Yoon et al., 2018). DeepQSM is trained on the synthetic data generated by the forward model and is validated on the *in vivo* dataset (Bollmann et al., 2019). Different kinds of convolutional kernels have also been employed to improve network performance, such as octave convolution (Gao et al., 2021). The algorithm unfolding is also introduced into CNN-based QSM, e.g., VaNDI (Polak et al., 2020). In addition, the proximal gradient descent algorithm is unrolled with CNN in LPCNN (Lai et al., 2020) and MoDL-QSM (Feng et al., 2021), in which COSMOS map and the component derived from susceptibility tensor imaging (STI; Liu, 2010) served as training labels. Recently, direct mapping from wrapped phase to magnetic susceptibility map has been implemented in iQSM (Gao et al., 2022), Affine transformation edited and refined deep neural network is used in AfterQSM (Xiong et al., 2023) for quantitative susceptibility mapping, and DeepSTI (Fang et al., 2023) implements STI with fewer orientations. However, the diversity of data distribution has not been well considered in existing works, leading to models with limited generalization ability in practical applications.

**Figure 1:**
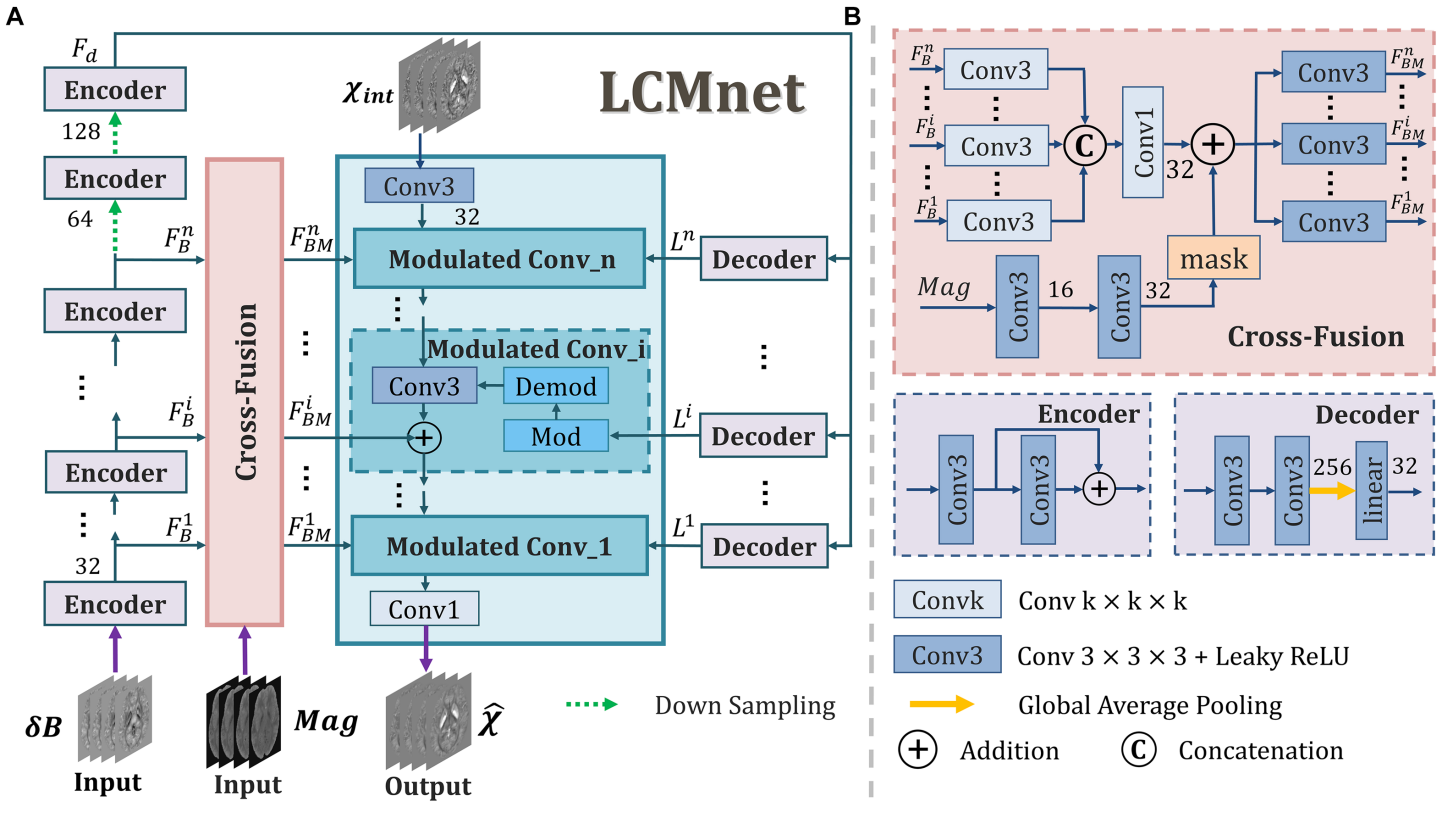
The architecture of our proposed LCMnet network. **(A)** The main framework includes the modulated convolution module, cross-fusion module, encoder and decoder blocks. **(B)** The detailed constitutions of some blocks.

In general, the field map is the single input of susceptibility reconstruction network, whereas the valuable information in magnitude images is not sufficiently explored. Actually, when tissue signals in the field map are contaminated by serious imaging artifacts, the magnitude image may provide some important guidance for network learning, e.g., to identify the clear morphology of lesions and blood vessels. In this work, we propose a latent code based modulation network with multi-variable fusion, named LCMnet, intending to achieve a more accurate susceptibility measurement. The multi-source data utilized in our network includes the field map, magnitude image and pre-estimated susceptibility data. Figure 1 illustrates the detailed architecture of LCMnet, where the modulated convolution module (MCM) is the network backbone. The latent code based modulated convolution enables the model to be robust to various data of different distributions. In experimental results, the brain data of healthy human, QSM challenge and clinical hemorrhage are used to demonstrate the method performance.

## 2 Methods

In this section, the ill-posed inversion problem in susceptibility reconstruction is first described, and then the details of our network designs are elaborated.

### 2.1 Susceptibility pre-estimation

In magnetic resonance imaging, a biological tissue with a magnetic susceptibility value will generate a field perturbation when it is located in a static field (Li and Leigh, 2004). As described in Eq. (1), the total induced perturbations *B*^∆^(*r*) under the static field are described by a convolution of the magnetic susceptibility *χ*(*r*) with the *z*-component $d_{z}\left(r\right)$ of the spatial unit dipole (Fang et al., 2019).


(1) $$B^{\Delta }\left(r\right)=\left|B_{0}\right|.\int \chi \left(r^{\prime }\right)\cdot d_{z}\left(r-r^{\prime }\right)d^{3}r^{\prime }$$

Where r and $r^{\prime }$ refer to the locations of the observed field and susceptibility source respectively, and $d_{z}\left(r\right)$ is defined as Eq. (2):


(2) $$\begin{array}{c} d_{z}\left(r\right)=\frac{3\cos^{2}\theta -1}{4\pi {\left|r\right|}^{3}} \end{array}$$

Where *θ* is the angle between r and *B*_0_. As described in Eq. (3), the magnetic field shift in the laboratory frame can be formulated as a point-wise multiplication in *k*-space:


(3) $$\begin{array}{c} \delta B\left(k\right)=\left(1/3-k_{z}^{2}/k^{2}\right)\cdot \chi \left(k\right) \end{array}$$

Where *χ* is the susceptibility distribution and k is the spatial frequency vector [$k_{x}$, $k_{y}$, $k_{z}$] with $k=\sqrt{k_{x}^{2}+k_{y}^{2}+k_{z}^{2}}$. Denoting the dipole kernel in the Fourier domain with $D_{k}\left(k\right)=1/3-k_{z}^{2}/k^{2}$ (Bao et al., 2021), the susceptibility map may be estimated from the measured field map using the inverse of the kernel as Eq. (4):


(4) $$\begin{array}{c} \chi_{k}\left(k\right)=D_{k}{\left(k\right)}^{-1}\delta B_{k}\left(k\right) \end{array}$$

This problem is ill-posed because of the zero values of $D_{k}\left(k\right)$ in *k*-space on a double conical surface at the magic angle of 54.7° (Bao et al., 2016). A straightforward approach is to perform a threshold based *k*-space division, i.e., the TKD approach (Wharton et al., 2010). As shown in Eq. (5), *k*-space masking was used to avoid noise amplification in regions where the kernel function is small:


(5) $$\begin{array}{c} \chi_{k}\left(k\right)=\{\begin{array}{c} \begin{array}{l} \delta B_{k}\left(k\right)/D_{k}\left(k\right),\left|D\left(k\right)\right|>T \end{array}\\ \;0,\left|D\left(k\right)\right|<T \end{array} \end{array}$$

Where *T* is the threshold value set to be 0.15–0.2, and k-space values in the ill-conditioned area are set to be zero in our experiments. In LCMnet model, we apply the susceptibility map pre-estimated by TKD method as one of the input sources, denoted as $\chi_{\mathrm{int}}$ in Figure 1A. This susceptibility pre-estimation is the input of the first modulated convolution block.

### 2.2 Feature extraction and multi-variable fusion

As depicted in Figure 1, a set of encoder and decoder blocks are connected in series and used as the basic units of LCMnet to acquire intermediate feature maps from the field maps. The feature maps gained by the *n* encoder blocks are denoted as {$F_{B}^{1}$,…,$\;F_{B}^{n}$}, which are sent to the cross-fusion block to fuse with the feature extracted from magnitude image. Furthermore, $F_{B}^{n}$ passes through two additional encoder blocks with downsampling, producing $F_{d}$ fed to the decoder block. The configurations of each encoder and decoder block are illustrated in Figure 1B. One decoder block consists of a convolutional layer, a global average pooling and a linear layer. It can convert the feature map $F_{d}$ into a latent code that reflects the distinctive attributes of feature maps. Considering that the background outside the volume of interest (VOI) differs in the training data, the operation of global average pooling is just restricted to the VOI.

The mechanism of multi-level features fusion makes the extracted features to be fully utilized and is conducive to enhancing the model capacity. When the information of different sources is fused effectively, the final reconstruction can be more reliable than only applying a single data source. In this way, the network can achieve better accuracy and robustness. We can see in Figure 1B that the cross-fusion block combines the multi-level feature maps learned from the field data with those extracted from magnitude images. In detail, the feature maps of each level $F_{B}^{i}$ are processed by one Conv3, and then all multiple levels are fused through a Conv1. After that, the fused feature maps go through a Conv3 and output feature maps {$F_{BM}^{1}$,…,$\;F_{BM}^{n}$}, which are one of the three inputs of each modulated block. In addition, the magnitude data used in the cross-fusion block is handled by a corresponding VOI mask, as shown in the inset of Figure 1B, to avoid the interference from the background information outside VOI.

### 2.3 Latent code based modulation

The modulated convolution is an operation that multiplies the parameters of the convolution kernel with a specific modulating signal. For a generative model, the modulating signal can be the latent code carried by input images. In the literatures of Karras et al. (2020) and Yang et al. (2021), by varying values of the latent code, it is possible to control multiple attributes of the generated image, thereby modifying the data distribution. Since the field maps will have different intensity scales with respect to organs, acquisition settings and so on, we introduce a latent code based modulation to enable the model auto-adaption to input data distribution. In Figure 1A, the modulated block is denoted as Modulated Conv_i, encompassing both modulation and demodulation operations, separately denoted as Mod and Demod. In our LCMnet, the MCM is constituted by three modulated blocks. Each of them is composed of three inputs: feature maps extracted from the susceptibility data, the latent code learned from the field data, and the multi-level feature maps produced from fused information of magnitude and field data. As the initial input of the MCM, the susceptibility map pre-estimated from TKD provides a raw approximation, potentially reducing the complexity of the model while increasing its stability and convergence speed. Within modulated convolution blocks, the latent code of the field maps is utilized to modulate the parameters of the convolution kernels, and the modulation operation can be expressed by the following equation:


(6) $$\begin{array}{c} w_{p,q,r,c}^{\prime }=l_{c}.w_{p,q,r,c} \end{array}$$

Where $w_{p,q,r,c}$ and $w_{p,q,r,c}^{\prime }$ represent the original and modulated parameters of the convolution kernel. The subscripts of p,q,r indicate the coordinates of the convolution kernel. $L^{i}$ is a vector representing the latent code, whose length is the same as the number of total channels. $L^{i}=\left\{l_{1},\ldots l_{c},\ldots l_{\text{total\_channels}}\right\}$, where *i* represent the index of modulated convolution block in Figure 1A, and $l_{c}$ is the corresponding latent code element for channel c. Accordingly, the learned latent codes can adjust the model parameters so that the modulated convolution is sensitive to the input data distribution. Subsequently, the demodulation operation normalizes the convolutional kernel to eliminate the numerical bias caused by the intensity information and to make model training more stable (Salimans and Kingma, 2016). This normalization process can be expressed in the form of:


(7) $$\begin{array}{c} w_{p,q,r,c}^{\prime \prime }=\frac{w_{p,q,r,c}^{\prime }}{\sqrt{\sum_{p,q,r,c}w_{p,q,r,c}^{\prime }{\;}^{2}+\varepsilon }} \end{array}$$

Where $w_{p,q,r,c}^{\prime \prime }$ is the parameter of the convolutional kernel after normalization and $w_{p,q,r,c}$ is the parameter of the convolutional kernel after being modulated by latent codes. A smaller positive constant ε is used to avoid zero division. Eqs. (6, 7) correspond to the operations of Mod and Demod in the modulated convolution shown in Figure 1. With the operation of Eqs. (6, 7), the latent codes can adjust the parameter of convolution kernel flexibly, making network adaptive to various susceptibility imaging.

## 3 Experiment

### 3.1 Data preparation

A set of volumetric data containing 44 phase measurements constitutes the *in vivo* healthy dataset. These volumetric data were acquired from a 7 T scanner with 32 channels head coil on ten healthy subjects with parameters of a voxel size = 1 × 1 × 1 mm^3^, FOV = 224 × 224 × 110 mm^3^, TR/TE1/∆TE = 45/2/2 ms, 5 ~ 9 echoes. This dataset is from LPCNN^1^ and the study is IRB approved with informed consent signed by each subject. Multiple steps of phase preprocessing were conducted on the *in vivo* dataset, including brain masking with FSL BET (Smith, 2002), phase unwrapping with a Laplacian-based method (Schofield and Zhu, 2003), and background field removal with iRSHARP (Fang et al., 2019). The multi-orientation data were registered to the supine position using FSL FLIRT (Jenkinson and Smith, 2001). After the coregistration, susceptibility maps were generated by COSMOS (Liu et al., 2009) as the gold standard in experiments. For COSMOS algorithm, maximum number of iterations is set to 200, tolerance is set to 10^−5^. Due to the limit of computational resources, the whole volume data was cropped into patches of size 64 × 64 × 32 during the network training process. In total, 6,860 pairs of cropped patches were applied for model training and eight volumetric data were used in the test.

In addition, the hemorrhage data was acquired using a 3 T MRI scanner with TE1/∆TE = 3.6/5 ms, FOV = 256 × 256 × 128 mm^3^, and matrix size = 240 × 240 × 64. This data is used to verify the clinical application performance, and those phase preprocessing steps were the same as the pipeline for the *in vivo* dataset. IRB approved informed consent was signed by the subject.

The 2016 QSM challenge data was a healthy human brain subject with twelve orientations and acquired using GRE sequences with wave-CAIPI at a resolution of 1.06 mm, TR/TE = 35/25 ms (Langkammer et al., 2018). The STI component $\chi_{33}$ was used as ground truth. The 2019 QSM challenge released one sample of simulated phantom with a calcification lesion, which is constructed based on a combination of *in vivo* T1 and T2^*^ brain maps (Bilgic et al., 2021). The images were acquired using a 7 T MRI scanner with TR/TEs = 50/4/12/20/28 ms, 0.64 mm isotropic resolution, and flip angle 15°. We employ this data to evaluate the generalization performance of LCMnet.

**Figure 2:**
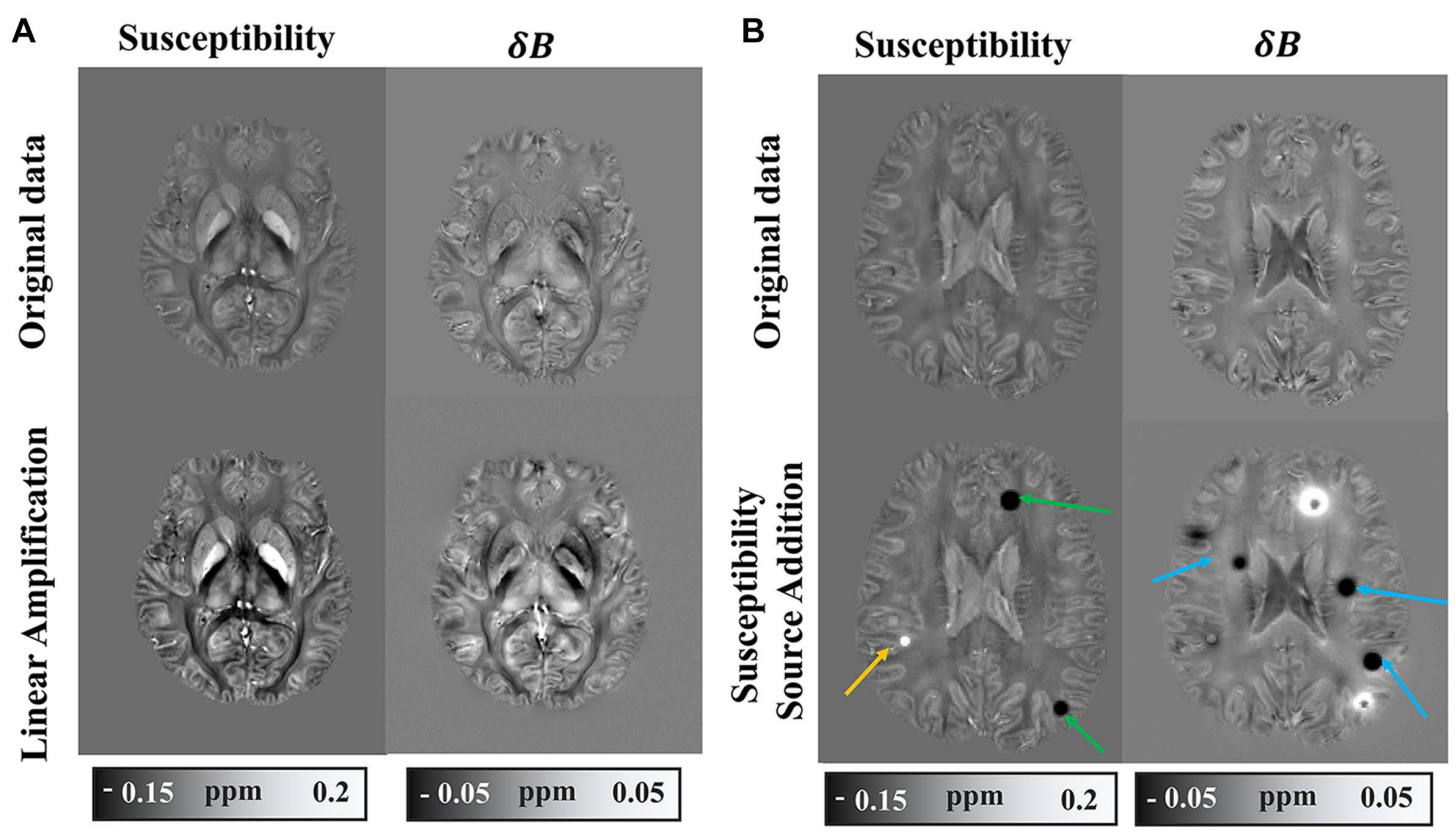
Example of synthetic dataset. **(A)** Synthetic data with numerical linear amplification operation. **(B)** Synthetic data with magnetic susceptibility source addition.

In order to diversify the dataset, a synthetic dataset was derived from the *in vivo* data through global linear amplification and the additional susceptibility sources. The global linear amplification factors are randomly set from 1 to 3. We added spherical magnetic susceptibility sources to the randomly selected brain tissue area, and set the value range of the magnetic susceptibility source between −0.4 ppm and 0.4 ppm. The field map corresponding to the magnetic susceptibility map is generated by the forward model, followed by the addition of random noise. The comparison between the synthetic dataset and the original *in vivo* dataset is illustrated in Figure 2. Figure 2B shows the field map of one slice and the corresponding magnetic susceptibility map. The large-scale field map fluctuations caused by strong magnetic susceptibility sources in adjacent slices may still have a significant impact on the displayed slice, therefore, the magnetic susceptibility source in δB and the susceptibility map do not pixel-wisely match in Figure 2. Considering the difference between synthetic data and real data, we retrained all deep learning models on simulated data using the same training methods used for training on healthy human brain data. The synthetic dataset serves the purpose of testing the adaptability of the deep learning model to a spectrum of data types.

### 3.2 Network training

The loss function for network training is a combination of MSE loss and HFEN loss (Ravishankar and Bresler, 2010), while it can be expressed as Eq. (8):


(8) $$\text{Loss}=∥\hat{\text{Y}}-\text{Y}{∥}_{F}^{2}+\pm \cdot ∥f_{LoG}\left(\hat{\text{Y}}\right)-f_{LoG}\left(\text{Y}\right){∥}_{F}^{2}$$

where $\hat{Y}$ is the result predicted by LCMnet. Y is the label data for network training. $∥\cdot {∥}_{F}$ represents the Frobenius norm. The filter operation $f_{\text{LoG}}$ is with the Laplacian of Gaussian kernel. The hyper-parameter α is to balance the two loss terms.

In this work, network models were implemented using Python with Pytorch backend and were trained on an NVIDIA Titan X GPU, Intel Xeon CPU E5 2.10 GHz, 64 GB RAM. The network optimizer was AdamW (Loshchilov and Hutter, 2017), and the weight decay was set to 5 × 10^−4^, with an initial learning rate of 10^−4^. We trained the LCMnet for 30 epochs with 15 h. To reduce memory consumption, the rim of whole volume data was appropriately tailored to the size of 192 × 192 × 108, and thus the GPU processing time of LCMnet was 0.85 s in test. The source code has been made publicly available at https://github.com/JoeWillbe/LCMnet.

### 3.3 Network architecture analysis

To validate the effectiveness of main modules in the proposed network, ablation experiments were designed and performed on the *in vivo* brain dataset. The modulated convolution module was replaced by the vanilla convolutions, denoted as LCMnet_NoMod. Similarly, the cross-fusion block was removed from LCMnet, denoted as LCMnet_NoFusion. The initial susceptibility map $\chi_{\mathrm{int}}$ was displaced with the field map to investigate the effect of susceptibility pre-estimation, denoted as LCMnet_deltaB.

**Figure 3:**
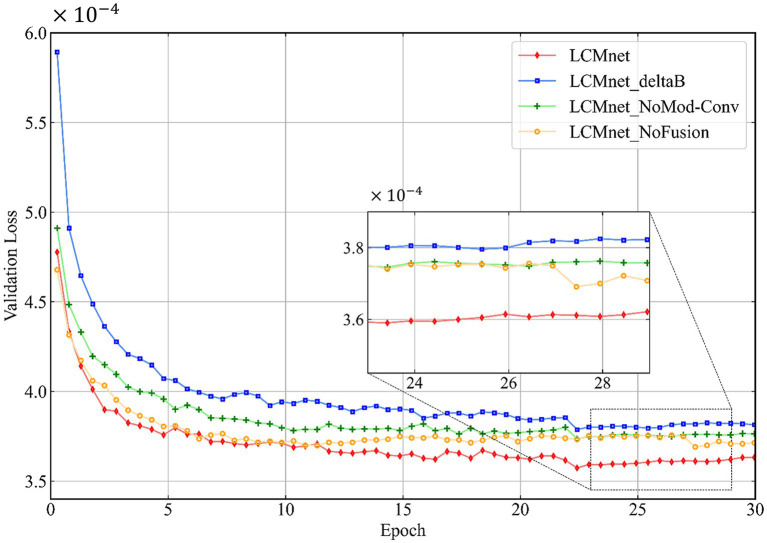
The network architecture analysis of LCMnet using convergence curves of the loss function.

We analyze the architecture of LCMnet using the convergence curves of the loss function, as shown in Figure 3. At the initial epochs, the loss of LCMnet_deltaB is significantly larger than other models, which demonstrates the effectiveness of susceptibility pre-estimation. In comparison, loss curves of LCMnet_NoMod and LCMnet_NoFusion are better than LCMnet_deltaB, but inferior to LCMnet. As listed in Table 1, the original LCMnet achieves the best scores on all evaluation metrics, indicating the success of each specific design.

**Table 1:** Quantitative comparisons of ablation experiments on the *in vivo* human brain dataset.

	PSNR	RMSE	MSSIM	HFEN
LCMnet_deltaB	37.48	50.44	0.935	56.41
LCMnet_NoMod	37.69	51.53	0.939	55.09
LCMnet_NoFusion	37.80	50.94	0.940	54.40
LCMnet	**37.91**	**53.70**	**0.946**	**53.70**

The bold values indicate that they are the best among various methods.

To assess the contribution of magnitude images, the input of magnitude data as shown in the inset of Figure 1B is deleted from the Cross-Fusion block. The modified model is denoted as LCMnet w/o Mag and is retrained by the *in vivo* dataset and then tested on the hemorrhage data. The result in comparison is illustrated in the Supplementary Figure S1. We can see that the susceptibility values of the hemorrhage region are underestimated in the LCMnet w/o Mag result. In contrast, the susceptibility map reconstructed by LCMnet presents more clear contours of the hemorrhage lesion, as indicated by the blue arrow, which implies that magnitude images may provide more valuable information for tissue structures.

## 4 Results

### 4.1 Healthy brain data

**Figure 4:**
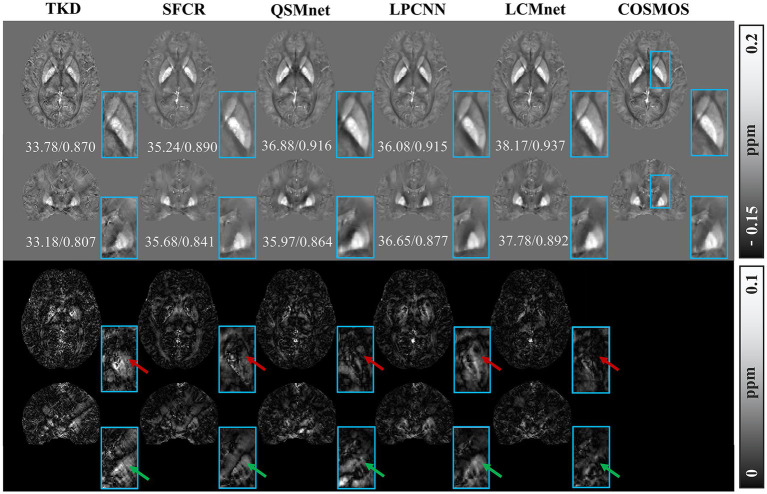
Experiment results of different methods on *in vivo* human brain data. The top two rows show the results of different QSM reconstruction methods, PSNR/MSSIM value annotation below the slice, and the bottom two rows show the difference maps between the reconstructed susceptibility maps and the COSMOS. The zooming views of deep gray matter are also given in the insets.

**Figure 5:**
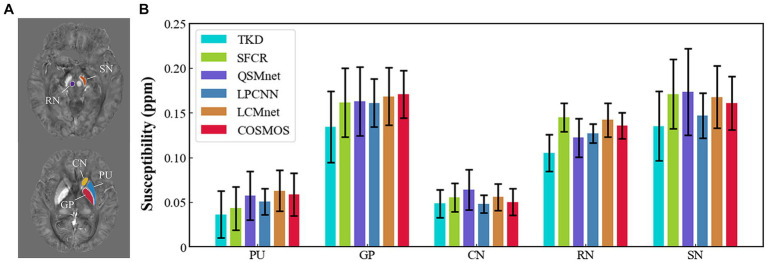
**(A)** ROI contours outlined in two different slices. **(B)** Bar graph compares the mean susceptibilities and standard deviations of ROIs in deep gray matter reconstructed by TKD, SFCR, QSMnet, LPCNN and LCMnet, taking the COSMOS values as the reference. Caudate nucleus (CN), globus pallidus (GP), putamen (PU), red nucleus (RN), and substantia nigra (SN).

Figure 4 shows the reconstructed results of the *in vivo* human brain data in axial and coronal views. The scores of PSNR and MSSIM are noted in the figure, while the arrows indicate areas with notable differences. The susceptibility maps generated from LCMnet are the closest to COSMOS and have the best PSNR/MSSIM. More details can be observed in the zooming views and difference maps. Table 2 lists the quantitative metrics of the results reconstructed by different methods, where each score is an average of eight samples. In accordance with Figure 4, LCMnet achieves the best results on all evaluation metrics, i.e., PSNR 37.15, RMSE 56.95, MSSIM 0.940 and HFEN 54.88. Figure 5 also presents the bar graph analysis of ROIs selected from the deep gray matter. In general, the susceptibility values of all nuclei produced by LCMnet are close to those of COSMOS, which may facilitate research on neurodegenerative diseases.

**Table 2:** Quantitative comparisons of the healthy brain results reconstructed by different methods.

	PSNR	RMSE	MSSIM	HFEN
TKD	34.93 ± 0.74	73.15 ± 5.97	0.921 ± 0.009	70.74 ± 7.33
SFCR	35.96 ± 1.02	65.69 ± 7.65	0.929 ± 0.010	57.77 ± 10.08
QSMnet	36.09 ± 0.69	64.19 ± 5.05	0.934 ± 0.006	58.61 ± 7.13
LPCNN	36.57 ± 1.05	60.89 ± 7.21	0.936 ± 0.009	58.49 ± 8.63
LCMnet	**37.15 ± 0.97**	**56.95 ± 6.26**	**0.940 ± 0.008**	**54.88 ± 6.27**

The bold values indicate that they are the best among various methods.

To confirm whether LCMnet can still perform better than other models on data obtained by tilted orientation, we compare all methods with reconstructions between the head supine position and a tilted one of the same subject, as given in Supplementary Tables S1, S2. It reveals that the results corresponding to the supine orientation are better than other tilted orientations. Nevertheless, our proposed method consistently outperforms traditional methods such as TKD and SFCR, as well as deep learning-based methods like QSMnet and LPCNN. Furthermore, we compared the model reconstruction results of magnitude and field maps for different echoes. In Supplementary Figure S2 and Supplementary Table S3, the model performance decline is evident when using single-echo data compared to multi-echo data input. Within a certain range of time differences between echoes, the reconstruction results for single-echo data show minimal variation.

### 4.2 QSM challenge data

**Figure 6:**
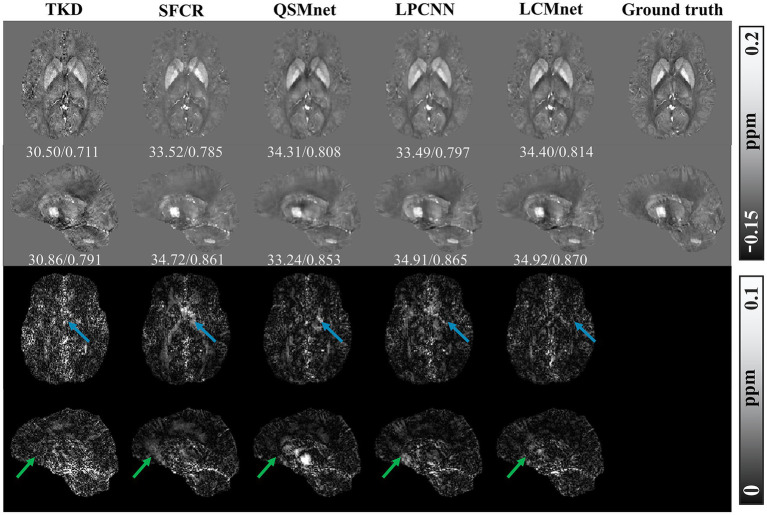
Experiment results of different reconstruction methods on the 2016 QSM challenge data, PSNR/MSSIM value annotation below the slice.

Figure 6 displays the susceptibility reconstructions from different methods on the 2016 QSM challenge data. As pointed out by the arrows, there are obvious structure errors in most of difference maps, but compared to other methods LCMnet exhibits less residual relative to the ground truth. For instance, TKD yields significant errors, and SFCR exhibits large differences over the junction of two hemispheres, meanwhile, QSMnet suffers from severe errors in the sagittal difference map. As for LPCNN, its results of susceptibility and difference maps are also not as good as LCMnet, especially in the deep gray matter region. Furthermore, the PSNR and MSSIM values of either axial view or sagittal view are the best, in agreement with the perceptual observation. Table 3 reports the reconstruction quantitative metrics of different methods, in which LCMnet achieves the highest PSNR of 34.56, the lowest RMSE of 70.95 and the highest MSSIM of 0.917. This result of LCMnet is generated by the model trained by the dataset acquired at 7 T MRI scanner without any retraining. If the network is fine-tuned using the 3 T data same with the 2016 QSM challenge data, the LCMnet results will be further improved.

**Table 3:** Quantitative results of different reconstruction methods on the 2016 QSM challenge data.

	PSNR	RMSE	MSSIM	HFEN
TKD	30.73	110.22	0.876	98.84
SFCR	34.30	73.11	0.915	**59.80**
QSMnet	34.38	72.43	0.914	68.51
LPCNN	34.55	71.04	0.913	68.18
LCMnet	**34.56**	**70.95**	**0.917**	70.77

The bold values indicate that they are the best among various methods.

**Figure 7:**
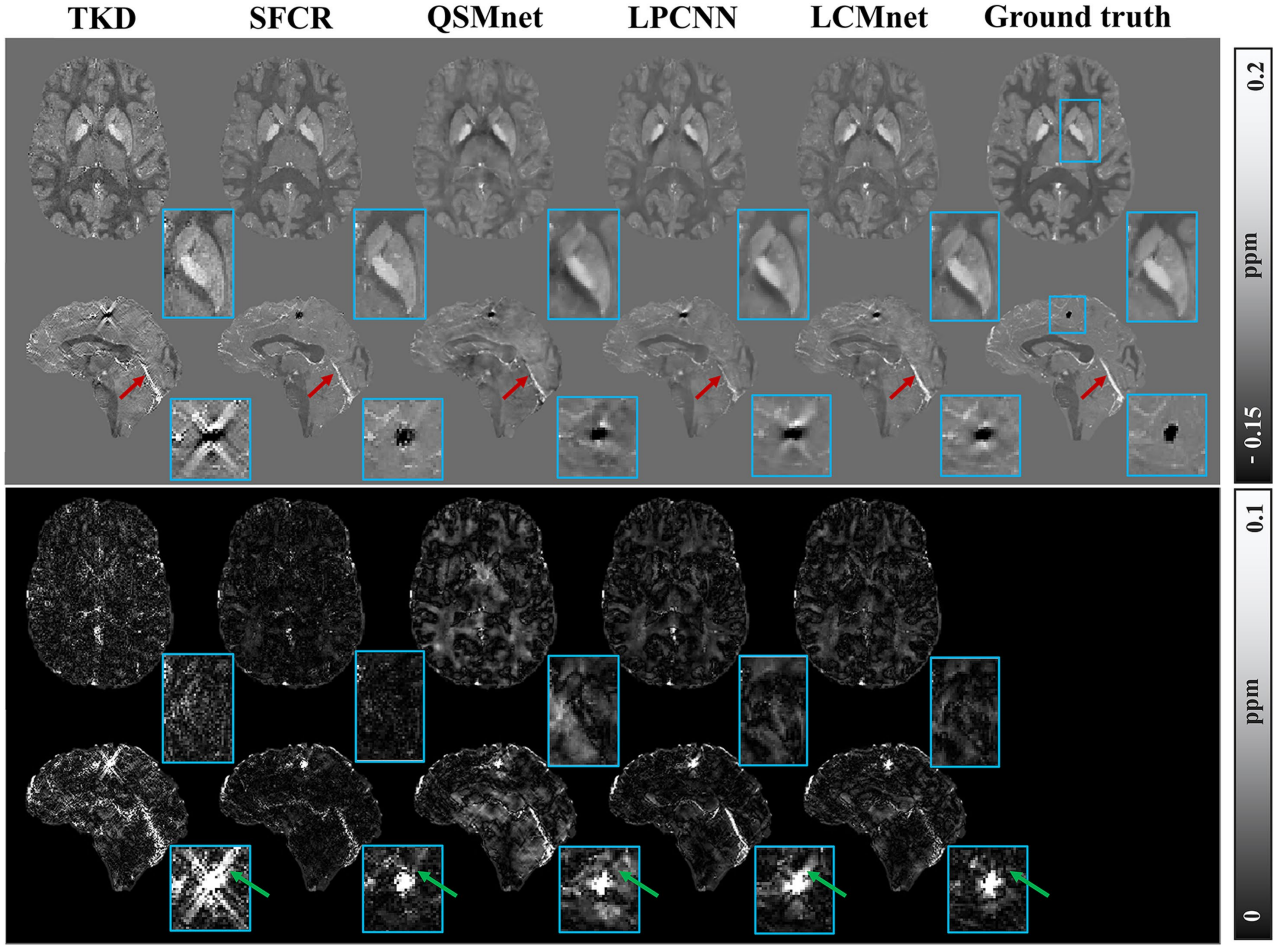
Experiment results of different reconstruction methods on the 2019 QSM challenge data, with zooming view of the calcified lesion on sagittal view.

With no fine-tuning, the model trained by the healthy brain data was further tested on the simulated phantom with calcification published by the 2019 QSM challenge, as shown in Figure 7. The TKD results show serious streaking artifacts around the calcified lesion, as indicated by the green arrow. The difference maps of SFCR have less error than QSMnet and LPCNN, and also the recovery of calcification is much better with no observable artifacts. Unfortunately, the results of QSMnet are poorer than SFCR, as there are many large residues in its difference maps. Although the reconstruction of LPCNN is improved compared to QSMnet, the results still contain some streaking artifacts in the region of calcified lesion. In all methods, LCMnet produces a good susceptibility map with better structure preservation. As pointed by the red arrows, the vessel structure in the LCMnet result is the clearest and the most coherent to ground truth. However, we should note that the network performance may be affected by image resolutions (Xiong et al., 2023). In this validation experiment, the LCMnet model is trained by data of 1 mm resolution, whereas the simulated phantom with calcification is in the resolution of 0.6 mm. If the model is retrained by data of the same resolution, the results can be further improved.

### 4.3 Hemorrhage data

**Figure 8:**
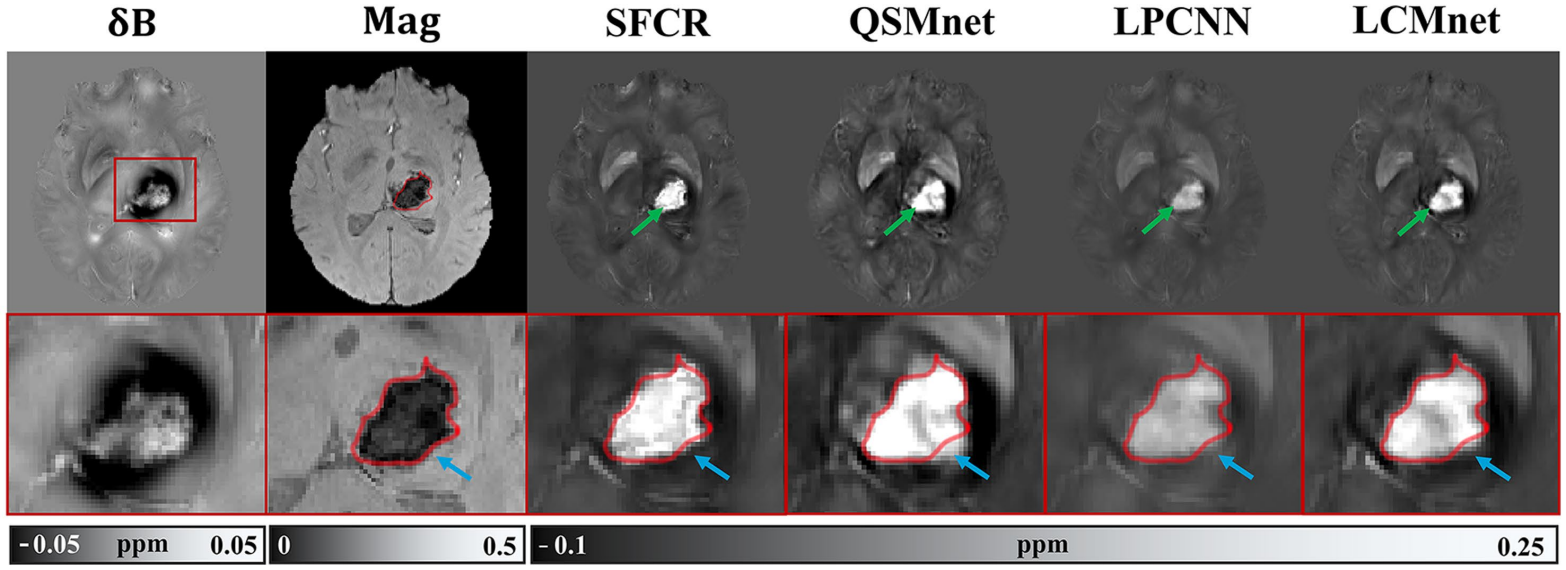
Experiment results of different methods on the hemorrhage data. The lesion area was extracted and shown in the zooming views. The lesion contour is marked by red curves.

We validate the generalization performance of LCMnet and its practical ability in clinical applications using a test on the hemorrhage data, as presented in Figure 8. Since the ground truth is unavailable for clinical data, the local field maps and magnitude images are displayed in the first and the second columns, where the hemorrhage area is enlarged in zooming views and the lesion contour is annotated by the red curve. We can observe in the field map that the susceptibility artifacts are so severe that the boundary of hemorrhage area is unable to be distinguished. On the contrary, the magnitude image presents a relatively clear hematoma contour.

Figure 8 compares the susceptibility maps reconstructed by the four methods, and they are unavoidably corrupted by certain image artifacts. The SFCR reconstruction can recover the hemorrhage with a contour similar to the magnitude image, where the voxels affected by noticeable susceptibility artifacts are not few. In the susceptibility map of QSMnet, the impact of artifacts is more serious than SFCR and the lesion boundary is rather inaccurate, but the internal hemorrhage contains some tiny structures. Meanwhile, LPCNN results have significantly lower contrast and yield relatively blurred tissue textures. In contrast, the susceptibility map reconstructed by LCMnet exhibits reasonable susceptibility values and moderate image artifacts, as well as fine tissue structures consistent with the magnitude image. This experiment result demonstrates that LCMnet possesses a good generalization ability that could be prospectively exploited with more clinical data.

### 4.4 Synthetic data

**Figure 9:**
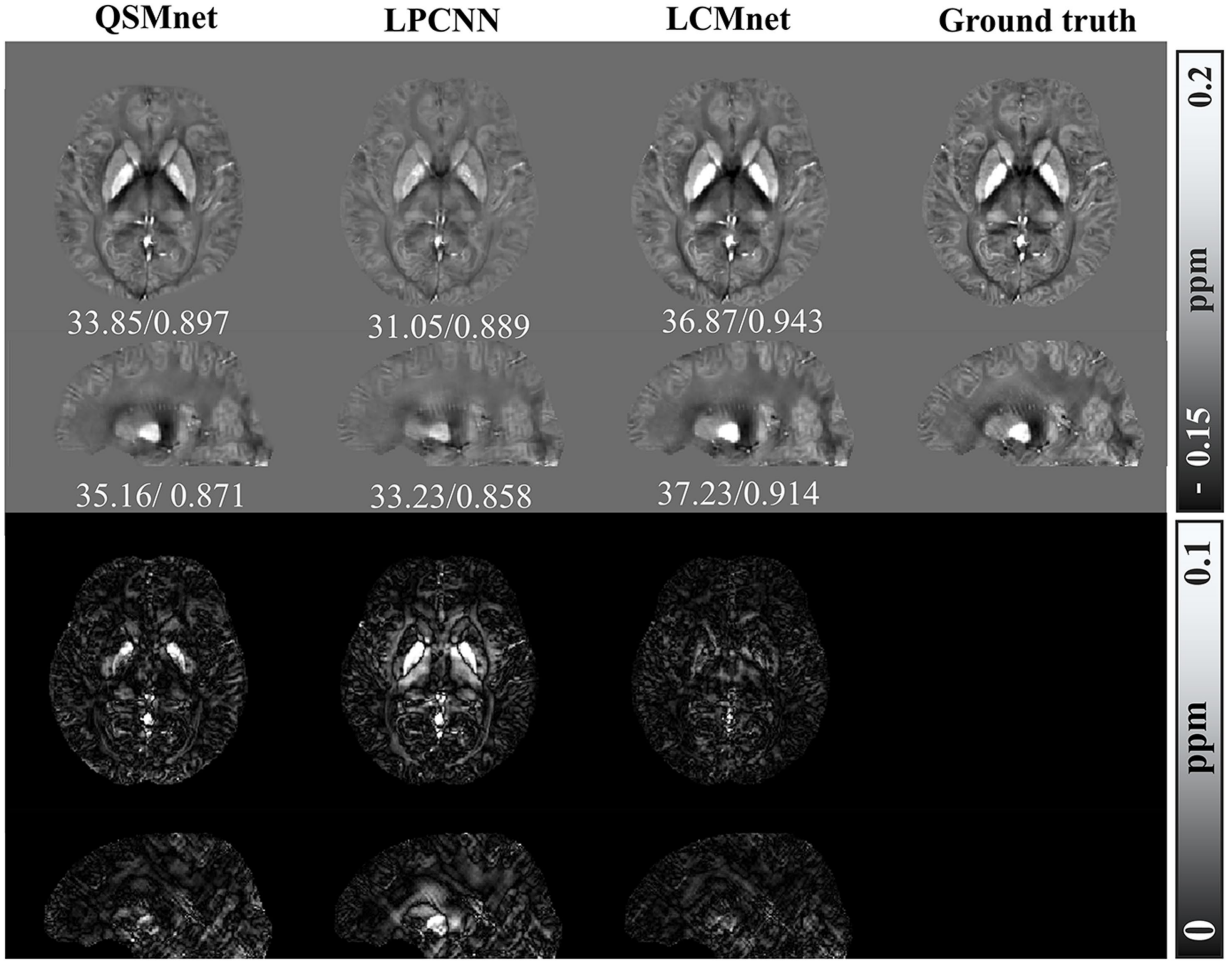
Comparison of reconstruction results of different models on numerical linear amplification data, PSNR/MSSIM value annotation below the slice.

The adaptability of deep learning-based QSM models to diverse datasets was evaluated through tests conducted on synthetic dataset. Test results for the synthetic data with susceptibility values linearly amplified are presented in Figure 9, with accompanying PSNR and SSIM values indicated below the visual representation. Notably, superior accuracy of reconstruction results over a wider range of susceptibility values are demonstrated by LCMnet. In contrast, QSMnet tends to underestimate magnetic susceptibility in the globus pallidus, and LPCNN exhibits excessive smoothing effects across the entire brain region.

**Figure 10:**
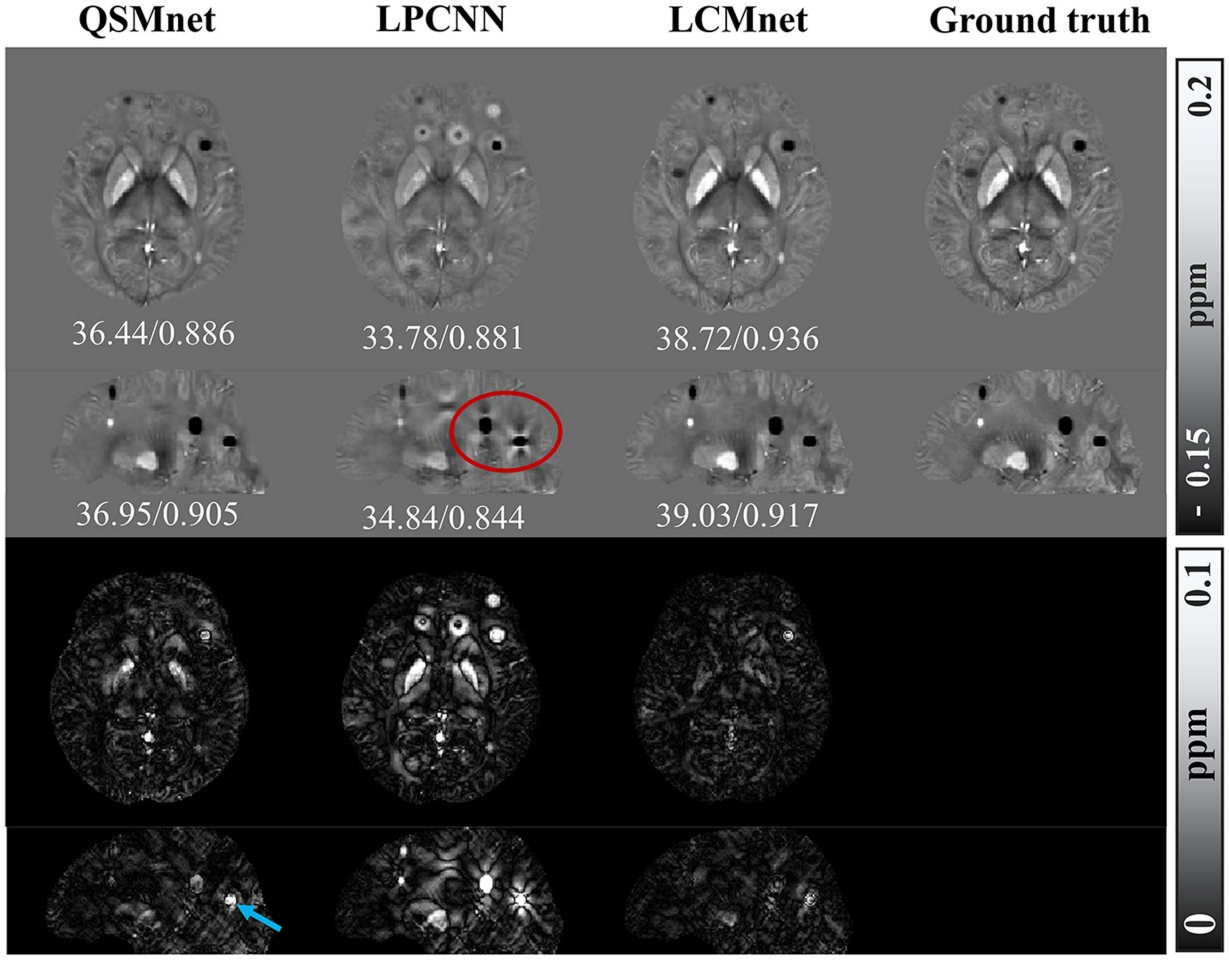
Comparison of reconstruction results of different models on data with additional susceptibility source, PSNR/MSSIM value annotation below the slice.

When processing synthetic data with additional susceptibility sources, as depicted in Figure 10, LCMnet is proven to accurately reconstruct susceptibility values for both simulated positive and negative magnetic susceptibility sources, effectively suppressing artifacts around each magnetic susceptibility source. While QSMnet can partially reconstruct magnetic susceptibility sources, it still exhibits some deviations in the reconstruction, as indicated by the blue arrows in the difference map. The susceptibility map reconstructed by LPCNN introduces pronounced cross-artifacts due to the presence of added magnetic susceptibility sources, as exemplified by the red circle in the figure. This substantial deviation can be attributed to interference from strong magnetic susceptibility sources in adjacent layers. The quantitative result comparisons are listed in Tables 4, 5 for synthetic data with linear amplification and additional susceptibility sources. It is observed that LCMnet achieved the most favorable reconstruction results compared to other methods.

**Table 4:** Quantitative results of deep learning QSM methods on the synthetic linear amplification data.

	PSNR	RMSE	MSSIM	HFEN
QSMnet	32.74 ± 3.14	52.18 ± 6.34	0.914 ± 0.018	49.88 ± 7.38
LPCNN	32.27 ± 3.24	55.01 ± 6.64	0.913 ± 0.021	56.04 ± 2.89
LCMnet	**34.53 ± 4.29**	**42.53 ± 6.64**	**0.939 ± 0.019**	**42.75 ± 6.44**

The bold values indicate that they are the best among various methods.

**Table 5:** Quantitative results of deep learning QSM models on the synthetic data with added susceptibility source.

	PSNR	RMSE	MSSIM	HFEN
QSMnet	35.57 ± 0.94	45.15 ± 9.11	0.921 ± 0.010	39.30 ± 8.39
LPCNN	33.55 ± 1.02	56.65 ± 2.78	0.924 ± 0.007	56.11 ± 7.04
LCMnet	**38.13 ± 1.37**	**33.62 ± 6.30**	**0.943 ± 0.009**	**30.34 ± 6.65**

The bold values indicate that they are the best among various methods.

## 5 Discussion

To validate the effectiveness of our designs in LCMnet, a group of ablation experiments were conducted. As the main backbone, modulated convolution module MCM explicitly incorporates the distribution information of the input data by the latent code based modulation. Specifically, the parameters modulation is performed on the channel dimension of convolution kernels and leads to an update of the feature map, according to latent codes learned from the decoder part. Once the network can appropriately respond to the input data with different distributions, it will be adaptive to adjust the intensity of feature maps during the network training. Attributable to the incorporation of MCM, our network demonstrates good generalization capabilities for the data with hemorrhage and calcification.

The inputs of LCMnet are composed of the field map, magnitude image and pre-estimated susceptibility data, forming a multi-variable fusion. The employment of multiple sources offers sufficient information to the MCM, thus bringing better accuracy to the model. The ablation result in Figure 3 reveals that when we take the pre-estimated susceptibility map as the initial input of MCM, it can dramatically reduce the initial loss of the model and accelerate convergence. In this work, the susceptibility pre-estimation is derived from a simple method of TKD that is easy to calculate, even so, this reasonable combination did boost the model performance. Despite some artifacts may exist in the pre-estimation, they are well suppressed by LCMnet in the final reconstruction, as the calcification data shown in Figure 7, which implies that the model is robust to any probable bias induced by the initial susceptibility map. On the other hand, the regions with large field fluctuations consistently exhibit severe serious susceptibility artifacts, such as blood vessels and lesions. It is impossible to obtain a precise reconstruction purely based on the field map. In this view, the magnitude images may provide complementary information for the network to discriminate the ground truth and artifacts. It has been demonstrated in Figure 8 and Supplementary Figure S1 that LCMnet can reconstruct a more accurate contour of the lesion with reasonable susceptibility values on the hemorrhage data. However, the other two networks of QSMnet and LCMnet w/o Mag yield blurred contours of the lesion area.

The latent code modulation technique allows the model to adaptively acquire information from multi-variable data. Results from synthetic datasets affirm that LCMnet exhibits robust adaptability to data with a broad numerical distribution range of susceptibility values. As the basic module to implement the modulation operation, the number of modulation convolution blocks used in MCM is 3. We also carried out an additional ablation experiment to discuss its proper number. In the Supplementary Table S4, a comparison of models with different numbers of modulated blocks in a range from 2 to 5 is given. This experiment reveals that the model performance is positively correlated with the block number. In detail, when setting the number to be 2, the quantitative metrics of the reconstructed healthy data are inferior. As the number of modulated convolution blocks in LCMnet is increased, a significant improvement is observed between 2 and 3, while the performances with 3 blocks and 4 blocks are comparable. Nonetheless, the number of model parameters also increases with more blocks. Therefore, we choose to utilize 3 modulated convolutional blocks in our experiments, which can be adjusted considering a trade-off between the computing cost and the reconstruction precision. In addition, given the portability of modulated convolution technology, this technique may be expected to enhance the accuracy and robustness of models in other image processing fields, such as STI.

Since the QSM reconstruction requires the network to realize an accurate end-to-end mapping from the local field data to the susceptibility value, we suggest not doing the normalization to the network input. Considering that LCMnet model used in all experiments is trained on the healthy human brain data, if the data with similar distribution is applied to retrain the network, the susceptibility results in Figures 6, 7 will be better. Accurate training label is necessary for a supervised learning strategy, but the medical imaging lacks ground truth, we employ COSMOS map as the substitution in this work. Normally, the network performance is sensitive to the training data, so the training dataset specifically tailored for the data in solution may promote the results. Our proposed method is validated on the brain MRI data, but its usage is not limited to the susceptibility measurement in brain imaging. It may provide an inspiring paradigm for researches related to quantitative measurements.

## 6 Conclusion

In this work, we proposed a latent code based modulation network with multi-variable fusion for QSM reconstruction. The latent code modulation technique enables the model to adaptively learn the information of multi-variable data. Taking the susceptibility pre-estimation as the network input, it endows an improvement in the model stability and training convergence. In addition, feature maps extracted from the field data are fused with magnitude features in the cross-fusion block, which further enhances the network capability for those challenging clinical data suffering from serious artifacts. In experiments, LCMnet was benchmarked against a suite of representative methods, i.e., the traditional TKD, the optimization-based SFCR, the deep learning based QSMnet and LPCNN, and the results show that LCMnet outperforms other methods in terms of measurement accuracy and practical generalization.

## Data availability statement

The original contributions presented in the study are included in the article/Supplementary material, further inquiries can be directed to the corresponding author.

## Ethics statement

The studies involving humans were conducted in accordance with the local legislation and institutional requirements, with informed consent from participants.

## Author contributions

WZ: Investigation, Methodology, Software, Validation, Writing – original draft, Writing – review & editing. JX: Investigation, Writing – review & editing. LB: Conceptualization, Data curation, Funding acquisition, Investigation, Methodology, Project administration, Resources, Supervision, Writing – review & editing.

## Footnotes

^1^ https://github.com/Sulam-Group/LPCNN
