# Supplementary material for: A latent code based multi-variable modulation network for susceptibility mapping
Source: Front Neurosci. 2023 Dec 21;17:1308829. doi: 10.3389/fnins.2023.1308829 (PMC10771344; doi:10.3389/fnins.2023.1308829)
Supplement: Supplementary file 1 [file Data_Sheet_1.docx]

Supplementary Material

# Supplementary Figures


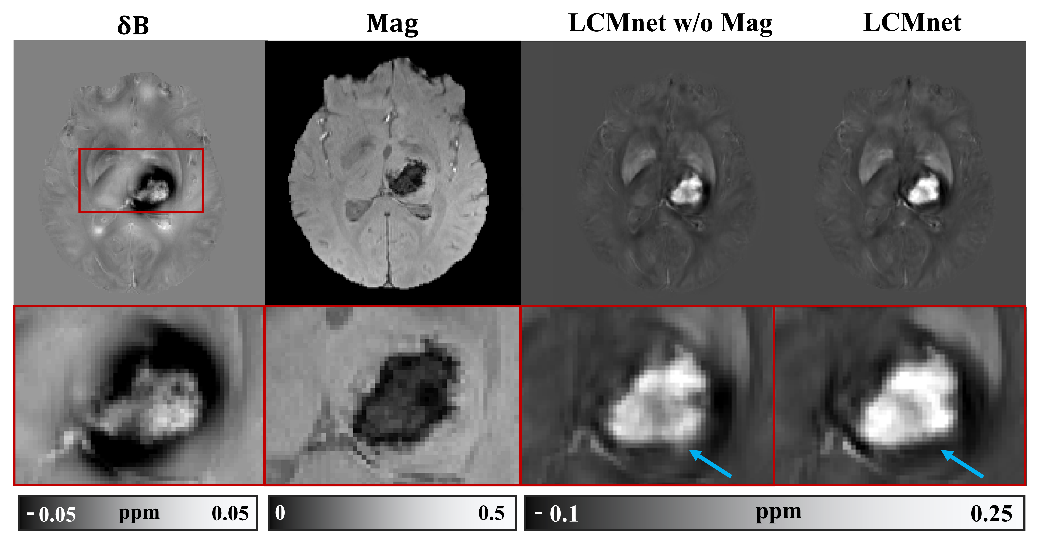


**Figure S1** Qualitative analysis of the ablation experiments on the effect of the magnitude map on the hemorrhage data.


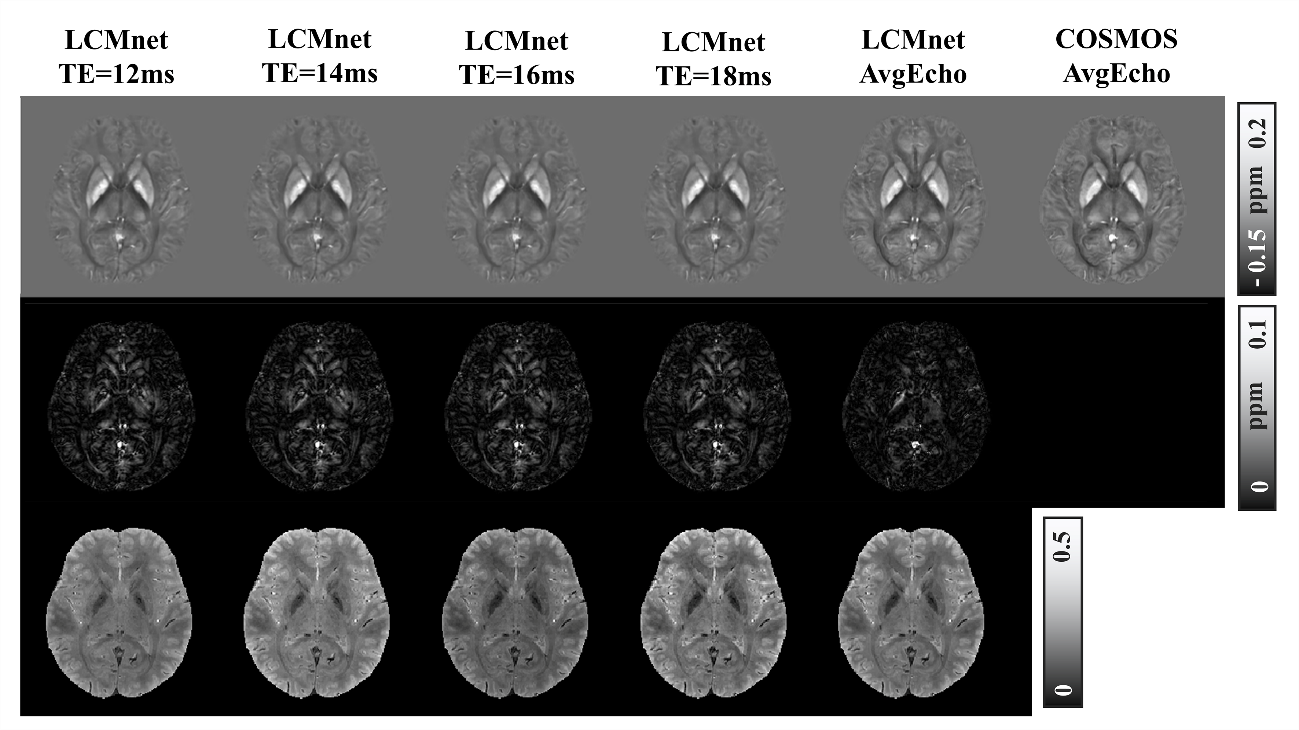


**Figure S2** A comparison on LCMnet results reconstructed by field and magnitude data of different echo times, i.e., TE={12,14,16,18}ms. In addition, susceptibility maps of LCMnet model and COSMOS are also presented here, where AvgEcho denotes the use of data averaged from multiple echoes. The second row represents the difference map between the susceptibility map of the first row and the COSMOS result. The third row shows the normalized magnitude maps for each echo.

# Supplementary Tables

**Table S1** Quantitative results on the supine orientation of the *in vivo* data using different methods.

|  | PSNR | RMSE | MSSIM | HFEN |
| --- | --- | --- | --- | --- |
| TKD | 35.10 | 69.44 | 0.927 | 65.89 |
| SFCR | 35.88 | 63.44 | 0.934 | 53.74 |
| QSMnet | 36.57 | 58.64 | 0.940 | 52.87 |
| LPCNN | 37.10 | 55.14 | 0.943 | 51.40 |
| LCMnet | **37.78** | **51.03** | **0.949** | **49.89** |

**Table S2** Quantitative results on the nonsupine orientation of the *in vivo* data using different methods.

|  | PSNR | RMSE | MSSIM | HFEN |
| --- | --- | --- | --- | --- |
| TKD | 33.80 | 80.66 | 0.905 | 75.70 |
| SFCR | 34.58 | 73.75 | 0.913 | 68.26 |
| QSMnet | 35.54 | 66.04 | 0.929 | 63.43 |
| LPCNN | 35.32 | 67.69 | 0.921 | 65.63 |
| LCMnet | **36.18** | **61.32** | **0.929** | **59.53** |

**Table S3** Quantitative comparisons of LCMnet using data with different echo time as input.

|  | PSNR | RMSE | MSSIM | HFEN |
| --- | --- | --- | --- | --- |
| TE=12ms | 36.66 | 61.01 | 0.9348 | 59.48 |
| TE=14ms | 36.67 | 60.92 | 0.9349 | 59.27 |
| TE=16ms | 36.68 | 60.86 | 0.9346 | 59.53 |
| TE=18ms | 36.67 | 60.90 | 0.9345 | 59.28 |
| AvgEcho | 37.78 | 51.03 | 0.9490 | 49.89 |

**Table S4** Quantitative comparisons of LCMnet models with different numbers of modulated convolution blocks.

|  | PSNR | RMSE | MSSIM | HFEN | Params (M) |
| --- | --- | --- | --- | --- | --- |
| n=2 | 37.45 | 56.66 | 0.939 | 53.31 | 3.769 |
| n=3 | 37.77 | 54.61 | 0.945 | 50.62 | 5.244 |
| n=4 | 37.84 | 54.14 | 0.946 | 59.06 | 6.719 |
| n=5 | **37.87** | **53.95** | **0.946** | **49.19** | **8.194** |
