# Supplementary figures and images for: A latent code based multi-variable modulation network for susceptibility mapping
Source: Front Neurosci. 2023 Dec 21;17:1308829. doi: 10.3389/fnins.2023.1308829 (PMC10771344; doi:10.3389/fnins.2023.1308829)

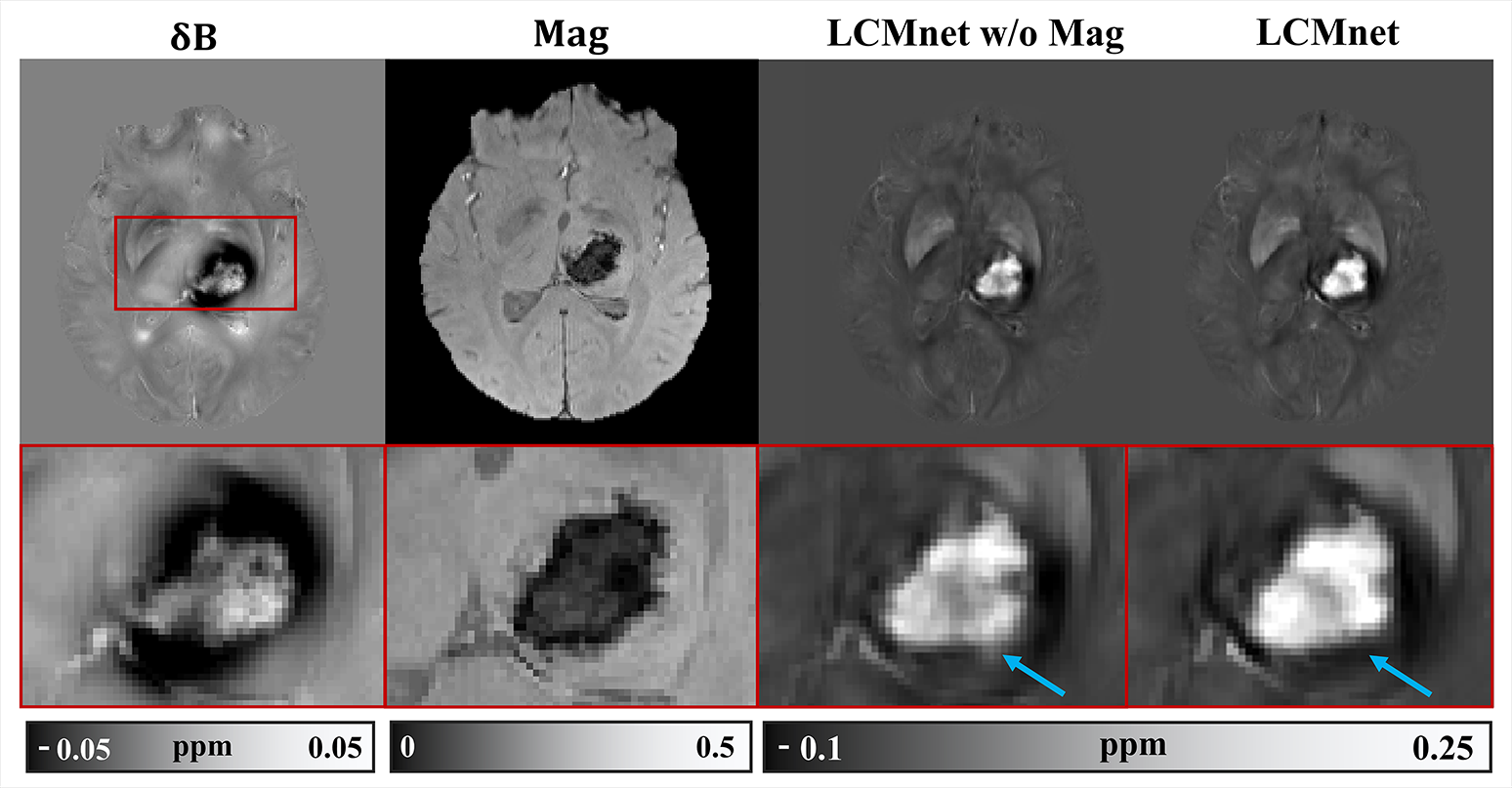

Supplement: Supplementary file 2 [file Image_1.TIF]

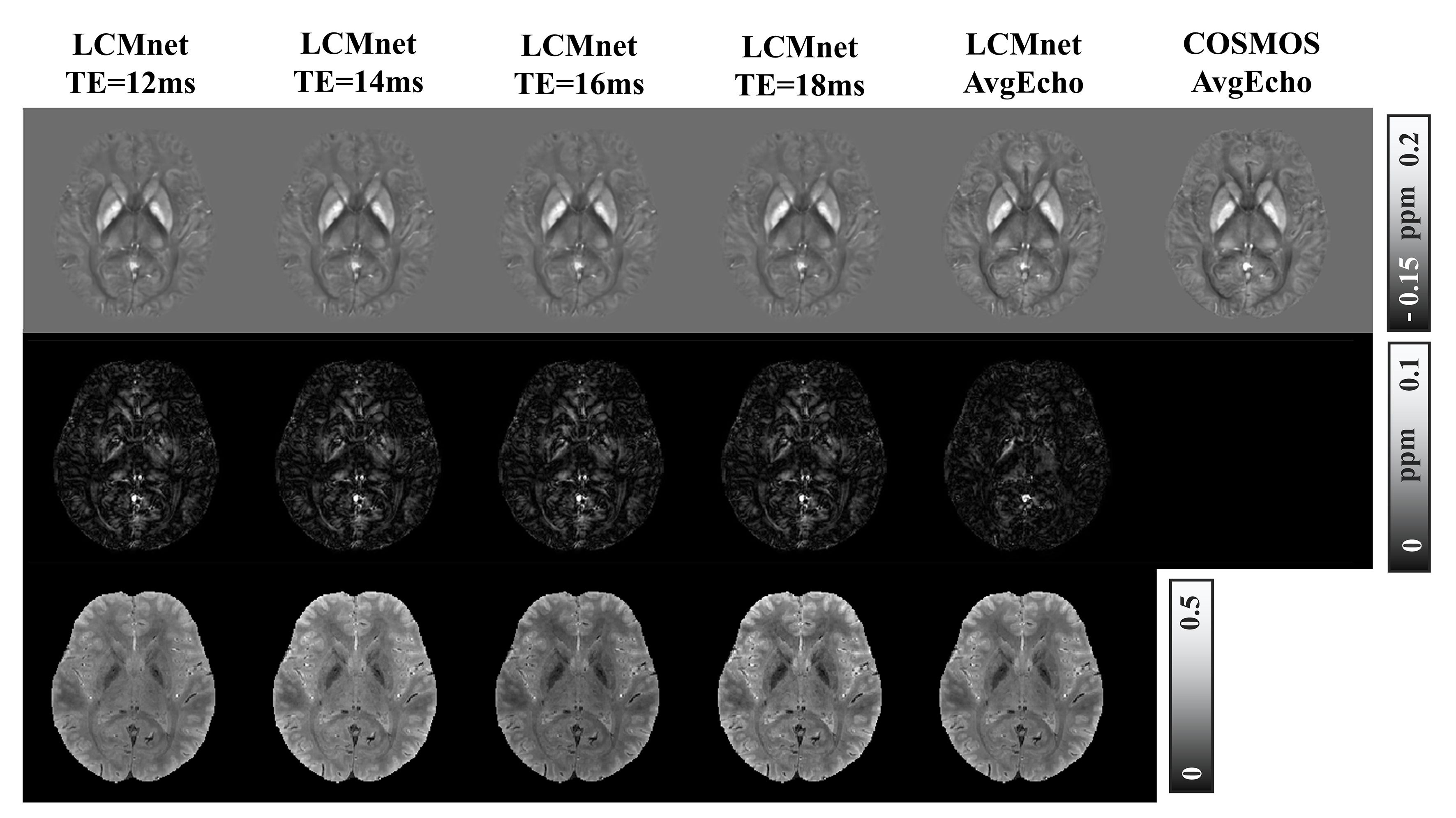

Supplement: Supplementary file 3 [file Image_2.TIF]
